# Clinical characteristics and risk factors of necrotizing pneumonia secondary to severe pneumonia in children

**DOI:** 10.3389/fped.2026.1837505

**Published:** 2026-05-20

**Authors:** Lin Che, Weifang Zhou, Qinghui Chen, Fangfang Cheng, Jianmei Tian

**Affiliations:** Department of Infectious Diseases, Children’s Hospital of Soochow University, Suzhou, China

**Keywords:** children, necrotizing pneumonia, prediction, risk factors, severe pneumonia

## Abstract

**Objective:**

To identify clinical risk factors and characterize associated features of necrotizing pneumonia (NP) in children with severe pneumonia.

**Methods:**

This retrospective case–control study included children with severe pneumonia hospitalized at the Children's Hospital of Soochow University between July 2022 and June 2025. Children who met the diagnostic criteria for NP were classified into the NP group, while a 1:2 control group (NNP) was randomly selected from eligible patients without NP during the same period. Clinical characteristics were compared between the two groups, followed by risk factor analysis and construction of ROC curves.

**Results:**

A total of 33 children with NP were included. Compared with the NNP group, significant differences were observed in duration of fever, hemoptysis, wheezing, chest pain, duration of corticosteroid use after admission, alanine aminotransferase, lactate dehydrogenase, albumin, white blood cell count (WBC), neutrophil percentage, platelet count, C-reactive protein, fibrin degradation products (FDPs), fibrinogen, D-dimer, anticoagulant therapy, pulmonary consolidation, pleural effusion, mucus plugs, and the number of bronchoscopic procedures (all *P* < 0.05). After variable selection using LASSO regression under the *λ*1se criterion, duration of fever, wheezing, chest pain, WBC, FDPs, and the number of bronchoscopic procedures remained associated with NP. Further multivariable logistic regression and ROC curve analyses demonstrated that chest pain, elevated WBC and FDPs were independent factors associated with NP. The combined model of these three variables showed good predictive performance for NP, with an AUC of 0.941 (95% CI: 0.891–0.990), and both sensitivity and specificity of 87.88%.

**Conclusion:**

Chest pain, elevated WBC, and increased FDPs were independently associated with NP, suggesting that these factors may help identify children at higher risk and inform clinical assessment.

## 1 Introduction

Necrotizing pneumonia (NP) is an infrequent but severe complication of community-acquired pneumonia (CAP) in children, characterized by necrosis, liquefaction, and cavitation of the pulmonary parenchyma (1). Histopathological studies show marked inflammatory infiltration, alveolar consolidation, and intrapulmonary vascular thrombosis, with accompanying necrotic areas and small cavities (2). NP predominantly affects children aged 2–5 years, and retrospective studies indicate that its incidence has increased over the past two decades, comprising up to 40% of cases of complicated pneumonia (3). Complicated pneumonia generally refers to pneumonia associated with parapneumonic effusion (PPE), empyema, NP, or lung abscess (4).

NP is characterized by extensive destruction and liquefaction of lung tissue and can occur even in patients receiving appropriate antimicrobial and anti-inflammatory therapy. The pathophysiology of NP remains incompletely understood and is likely multifactorial, involving impaired host defenses, infection with highly virulent or high-load pathogens, and perturbation of the pulmonary microbiome (5). Evidence indicates that NP may be associated with infection-induced vasculitis and endothelial injury, leading to coagulation abnormalities, vascular occlusion, and thrombosis. These processes ultimately result in ischemic injury, liquefaction, and necrosis of consolidated lung tissue (1, 5, 6). Clinically, children with NP often present with a severe disease course, including persistent high fever, respiratory distress, and extensive pulmonary consolidation on imaging and clinical assessment. A substantial proportion of patients develop complications, including parapneumonic effusion, empyema, bronchopleural fistula (BPF), sepsis, and respiratory failure (7). NP is also associated with prolonged hospitalization and increased mortality. Although current evidence suggests that most children with necrotizing pneumonia have favorable long-term outcomes, limited data indicate that mild pulmonary function changes may occur in some cases; therefore, follow-up evaluation may still be considered (3, 4, 8).

Despite increasing recognition of NP in recent years, early identification remains challenging, and reliable risk stratification tools are lacking in clinical practice. Identifying risk factors for NP in children with severe pneumonia and developing effective predictive models are therefore crucial for timely detection, early intervention, and optimized clinical management, ultimately improving patient outcomes. In this study, we retrospectively analyzed children with severe pneumonia to identify independent factors associated with NP and to assess the predictive utility of these indicators, providing evidence to support early risk assessment in clinical practice.

## 2 Methods

### 2.1 Study population

This was a single-center retrospective case–control study. Children aged ≥1 year who were hospitalized with newly diagnosed severe pneumonia at the Children's Hospital of Soochow University between July 2022 and June 2025 were enrolled. Patients with confirmed chronic lung disease, inherited metabolic disorders, chromosomal abnormalities, or immunodeficiency were excluded.

Patients who progressed to necrotizing pneumonia were classified as the NP group. Controls (non-NP, NNP) were randomly sampled at a 1:2 ratio from children with severe pneumonia during the same period who did not develop NP using computer-generated random numbers. These patients could have severe pneumonia-related findings, such as pulmonary consolidation or pleural effusion, but did not meet computed tomography (CT)-based criteria for NP. Other cavitary pulmonary diseases, including lung abscess, pulmonary tuberculosis, and congenital pulmonary airway malformation, were excluded during diagnostic classification.

The management of severe pneumonia in our department generally follows the Guidelines for the Management of Community-Acquired Pneumonia in Children (9), with individualized adjustments based on each patient's clinical condition. Specifically, glucocorticoids are primarily administered in children with a pronounced inflammatory response or evident disease progression. Anticoagulant therapy is considered for patients with markedly elevated D-dimer levels or an increased risk of thrombosis. Bronchoscopy is mainly indicated in cases of airway obstruction, mucus plugging, or atelectasis. In addition, for severe Mycoplasma pneumoniae pneumonia, glucocorticoids and, when necessary, anticoagulation may also be used as adjunctive therapies. These interventions are not applied routinely but are implemented on an individualized basis according to clinical assessment.

This study was approved by the Ethics Committee of the Children's Hospital of Soochow University (Approval No. 2024CS098, approved on August 2, 2024), and written informed consent was obtained from the guardians of all participants.

### 2.2 Diagnostic criteria

Severe pneumonia: Diagnosed according to the Guidelines for the Management of Community-Acquired Pneumonia in Children (2024 revision) (9).

Necrotizing pneumonia (NP): Diagnosed based on the following criteria: (1) Chest CT showing multiple air-filled cavities or thin-walled cavities without air–fluid levels within areas of pulmonary consolidation; (2) Exclusion of other cavitary lung diseases, including lung abscess, pulmonary tuberculosis, and congenital pulmonary airway malformation. The diagnosis of NP was established through a comprehensive assessment based on chest CT findings, in conjunction with clinical manifestations and the exclusion of other cavitary pulmonary diseases. To improve the reliability of diagnostic classification, CT images were independently and blindly reviewed by one pediatric physician and one radiologist. In cases of disagreement, a third senior physician adjudicated and determined the final diagnosis. As this was a retrospective study, no formal interobserver agreement analysis was further performed.

### 2.3 Data collection

Clinical characteristics, laboratory parameters, imaging findings, and treatment-related data were collected, including: (1) Clinical features: duration of fever, hemoptysis, wheezing, and chest pain; (2) Laboratory parameters: alanine aminotransferase (ALT), lactate dehydrogenase (LDH), albumin, white blood cell count (WBC), neutrophil percentage, platelet count (PLT), C-reactive protein (CRP), fibrin degradation products (FDPs), D-dimer, and fibrinogen (Fib); (3) Treatment-related variables: duration of corticosteroid use after admission, use of anticoagulant therapy, and number of bronchoscopic procedures; (4) Imaging and airway findings: chest CT features and the presence of mucus plugs observed during bronchoscopy. All laboratory and clinical variables, except outcome measures, were collected at the time of hospital admission and prior to the development of NP. Major surgical intervention was defined as thoracotomy, thoracoscopic surgery, decortication/debridement, or pulmonary resection.

### 2.4 Statistical analysis

Statistical analyses were performed using SPSS 27.0. Continuous variables with normal distribution were presented as mean ± standard deviation and compared using the independent-samples *t*-test; non-normally distributed data were expressed as median (P25, P75) and compared using the Mann–Whitney *U*-test. Categorical variables were presented as number (percentage) and analyzed using the *χ*^2^-test or Fisher's exact test. LASSO regression was applied for variable selection, with the optimal penalty parameter determined using the one-standard-error rule (*λ*_1se), representing the largest *λ* within one standard error of the minimum cross-validated error to produce a parsimonious and interpretable model. All laboratory and clinical variables, except outcome measures, were collected at hospital admission prior to NP development to ensure predictive validity and avoid data leakage. Variables selected by LASSO were then included in a multivariable logistic regression model to identify independent factors associated with NP, with ten-fold cross-validation (random seed 2022) used to assess model stability. FDPs were log-transformed prior to logistic regression analysis to reduce skewness, while original FDP values were used in ROC analysis for clinical interpretability. ROC curves were constructed to evaluate the predictive performance of individual variables and the combined model. A two-sided *P* value <0.05 was considered statistically significant.

## 3 Results

### 3.1 Baseline characteristics

A total of 99 children with severe pneumonia were included, comprising 33 in the NP group and 66 in the NNP group. In the NP group, there were 14 males and 19 females, with a mean age of 7.25 ± 3.40 years; in the NNP group, there were 28 males and 38 females, with a mean age of 5.95 ± 3.01 years.

Pathogen detection using bronchoalveolar lavage fluid was performed in all patients in the NP group. No definite pathogen was identified in 2 cases (6.06%). Mixed infections were observed in 54.55% of cases. Mycoplasma pneumoniae was detected in 26 cases (78.79%), including 13 cases of single-pathogen infection (39.39%). Streptococcus pneumoniae and Staphylococcus aureus were identified in 6 (18.18%) and 3 cases (9.09%), respectively. Radiologic complications in the NP group included atelectasis (n = 9), pulmonary consolidation (n = 32), pulmonary bulla (n = 1), pleural effusion (n = 18), pneumothorax (n = 2), and pulmonary hemorrhage (n = 2), compared with 15, 51, 1, 20, 2, and 1 cases, respectively, in the NNP group. No significant differences were observed between the groups in atelectasis, pulmonary bulla, pneumothorax, or pulmonary hemorrhage (all P > 0.05). All patients improved and were discharged after treatment, with continued outpatient follow-up. No deaths occurred; however, two patients in the NP group underwent limited non-surgical invasive procedures: one received closed thoracic drainage for massive pleural effusion, and the other underwent thoracentesis for pneumothorax.

### 3.2 Comparison of clinical characteristics between groups

The comparison of clinical data between the NP and NNP groups is presented in Table 1. No significant differences were observed in sex or age between the two groups (*P* > 0.05). In terms of clinical features and imaging findings, the NP group showed significantly higher rates of prolonged fever duration, hemoptysis, wheezing, chest pain, pulmonary consolidation, pleural effusion, and mucus plugs detected by bronchoscopy compared with the NNP group (all *P* < 0.05). Regarding laboratory parameters, levels of ALT, LDH, albumin, WBC, neutrophil percentage, PLT, CRP, FDPs, fibrinogen, and D-dimer were significantly higher in the NP group than in the NNP group (all *P* < 0.05). In addition, the NP group had a higher proportion of anticoagulant therapy, longer duration of corticosteroid use after admission, and a greater number of bronchoscopic procedures prior to NP development (all *P* < 0.05).

**Table 1:** Comparison of clinical characteristics between the NNP and NP groups.

Variables	NNP (*n* = 66)	NP (*n* = 33)	*P* value
Sex			1.000
Female (0)	38 (57.58%)	19 (57.58%)	
Male (1)	28 (42.42%)	14 (42.42%)	
Age (years)	5.95 ± 3.01	7.25 ± 3.40	0.055
Length of hospital stay (days)	9.00 (8.00, 13.00)	15.00 (11.00, 23.00)	<0.001
Duration of fever (days)	6.88 ± 3.97	11.24 ± 4.83	<0.001
Hemoptysis			0.011
No	66 (100.00%)	29 (87.88%)	
Yes	0 (0.00%)	4 (12.12%)	
Wheezing			0.046
No	41 (62.12%)	27 (81.82%)	
Yes	25 (37.88%)	6 (18.18%)	
Chest pain			<0.001
No	64 (96.97%)	20 (60.61%)	
Yes	2 (3.03%)	13 (39.39%)	
Duration of corticosteroid use after admission (days)	9.00 (8.00, 12.75)	15.00 (11.00, 23.00)	<0.001
ALT (U/L)	16.75 (12.55, 23.57)	38.10 (16.00, 93.50)	<0.001
LDH (U/L)	392.35 (319.70, 502.80)	450.30 (367.50, 862.00)	0.042
Albumin (g/L)	40.43 ± 4.04	36.38 ± 4.86	<0.001
WBC (×10^9^/L)	12.20 (9.64, 15.52)	17.81 (13.80, 21.06)	<0.001
Neutrophil percentage (%)	77.95 (65.03, 85.97)	85.80 (75.60, 89.50)	0.003
PLT (×10^9^/L)	407.76 ± 146.50	490.79 ± 157.75	0.011
CRP (mg/L)	23.77 (6.66, 46.64)	59.06 (31.60, 126.42)	<0.001
FDPs (*μ*g/L)	2,905.00 (2,430.00, 4,920.00)	10,340.00 (4,370.00, 14,290.00)	<0.001
D-dimer (μg/L)	715.00 (430.00, 1,537.50)	2,940.00 (1,750.00, 5,230.00)	<0.001
Fibrinogen (g/L)	4.39 (3.87, 5.33)	5.77 (4.44, 6.85)	0.012
AT-III (%)	115.00 (107.00, 122.75)	116.00 (104.00, 123.00)	0.961
Anticoagulant therapy			<0.001
No	56 (84.85%)	16 (48.48%)	
Yes	10 (15.15%)	17 (51.52%)	
Pulmonary consolidation			0.012
No	15 (22.73%)	1 (3.03%)	
Yes	51 (77.27%)	32 (96.97%)	
Pleural effusion			0.019
No	46 (69.70%)	15 (45.45%)	
Yes	20 (30.30%)	18 (54.55%)	
Mucus plugs			0.015
No	50 (75.76%)	17 (51.52%)	
Yes	16 (24.24%)	16 (48.48%)	
Number of bronchoscopic procedures	1.00 (1.00, 2.00)	2.00 (2.00, 3.00)	<0.001

NNP, non-necrotizing pneumonia; NP, necrotizing pneumonia; ALT, alanine aminotransferase; LDH, lactate dehydrogenase; WBC, white blood cell count; PLT, platelet count; CRP, C-reactive protein; FDPs, fibrin degradation products; AT-III, antithrombin III.

### 3.3 Risk factor selection and multivariable analysis

**Figure 1:**
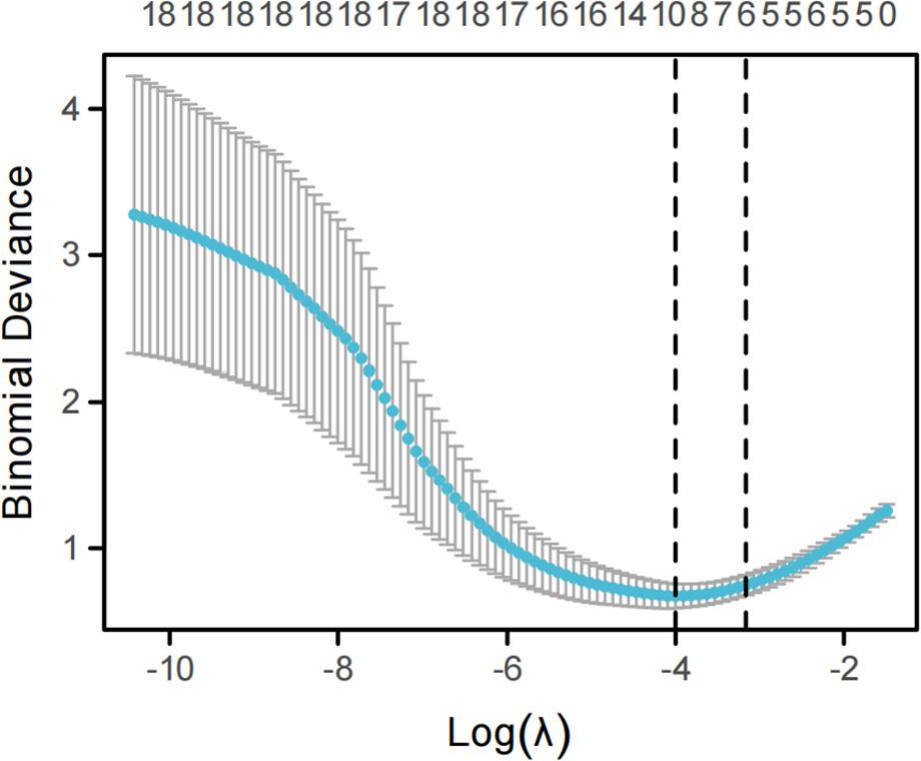
Variable selection using LASSO regression for necrotizing pneumonia (NP). The lower *x*-axis shows log(*λ*). The numbers on the upper *x*-axis indicate the number of nonzero coefficients corresponding to each *λ*. The *y*-axis represents the deviance or C-index under different metrics. Points denote the mean deviance for each *λ* during cross-validation, and vertical bars (error bars) indicate the standard error of the deviance for each *λ*. The left dashed line marks the optimal *λ* (λ_min), while the right dashed line marks *λ*_1se, which is the largest *λ* within one standard error of the minimum cross-validated error, selected to yield a simpler and more interpretable model while maintaining near-optimal predictive performance.

Based on prior clinical experience and evidence from previous studies, variables with potential clinical relevance were preselected, and LASSO regression was applied to identify factors associated with NP prior to its development. Using the *λ*1se criterion, six candidate variables were retained: duration of fever, wheezing, chest pain, WBC, Log_FDPs, and the number of bronchoscopic procedures (Figure 1 and Table 2). These variables were subsequently entered into a multivariable logistic regression model (Table 3), with NP occurrence as the dependent variable. The results demonstrated that chest pain (OR = 189.540, 95% CI: 9.649–3,723.336, *P* < 0.001), WBC [OR = 1.243, 95% CI: (1.070–1.452), *P* = 0.006], and Log_FDPs (OR = 509.594, 95% CI: 10.776–24,048.002, *P* = 0.002) were independently associated with NP.

**Table 2:** Variable selection for necrotizing pneumonia using LASSO regression.

**Variables**	***λ*min**	***λ*1se**
(Intercept)	−5.7656	−4.0403
Duration of fever	0.1086	0.0973
Hemoptysis	0	0
Wheezing	−0.6797	−0.1690
Chest pain	2.6347	1.9010
Duration of corticosteroid use after admission	0	0
ALT (U/L)	0	0
LDH (U/L)	0	0
Albumin (g/L)	0	0
WBC (×10^9^/L)	0.1028	0.0662
Neutrophil percentage (%)	0.0001	0
PLT (×10^9^/L)	0.0006	0
CRP	0	0
Log_FDPs	0.0002	0.0001
Fibrinogen	0	0
D-dimer	0	0
Anticoagulant therapy	0	0
Pulmonary consolidation	0.0203	0
Pleural effusion	0	0
Mucus plugs	−0.2723	0
Number of bronchoscopic procedures	0.4622	0.3343

ALT, alanine aminotransferase; LDH, lactate dehydrogenase; WBC, white blood cell count; PLT, platelet count; CRP, C-reactive protein; FDPs, fibrin degradation products. Variables with non-zero coefficients at λ1se were considered selected for subsequent multivariable analysis.

**Table 3:** Multivariable logistic regression analysis of factors associated with necrotizing pneumonia based on LASSO-selected variables.

**Variables**	**Univariate analysis OR (95% CI)**	***P* value**	**Multivariate analysis OR (95% CI)**	***P* value**
Duration of fever	0.787 (0.696–0.889)	<0.001	0.829 (0.633–1.086)	0.173
Wheezing	2.744 (0.995–7.570)	0.051	5.696 (0.400–81.105)	0.199
Chest pain	20.800 (4.323–100.087)	<0.001	189.540 (9.649–3,723.336)	<0.001
WBC (×10^9^/L)	1.111 (1.041–1.192)	0.003	1.243 (1.070–1.452)	0.006
Log_FDPs	392.902 (36.804–4,206.603)	<0.001	509.594 (10.776–24,048.002)	0.002
Number of bronchoscopic procedures	0.256 (0.127–0.518)	<0.001	0.559 (0.160–1.949)	0.361

Variables included in the multivariable model were selected using LASSO regression (λ1se). OR: odds ratio; CI: confidence interval; WBC: white blood cell count; FDPs: fibrin degradation products.

### 3.4 Predictive performance analysis

**Figure 2:**
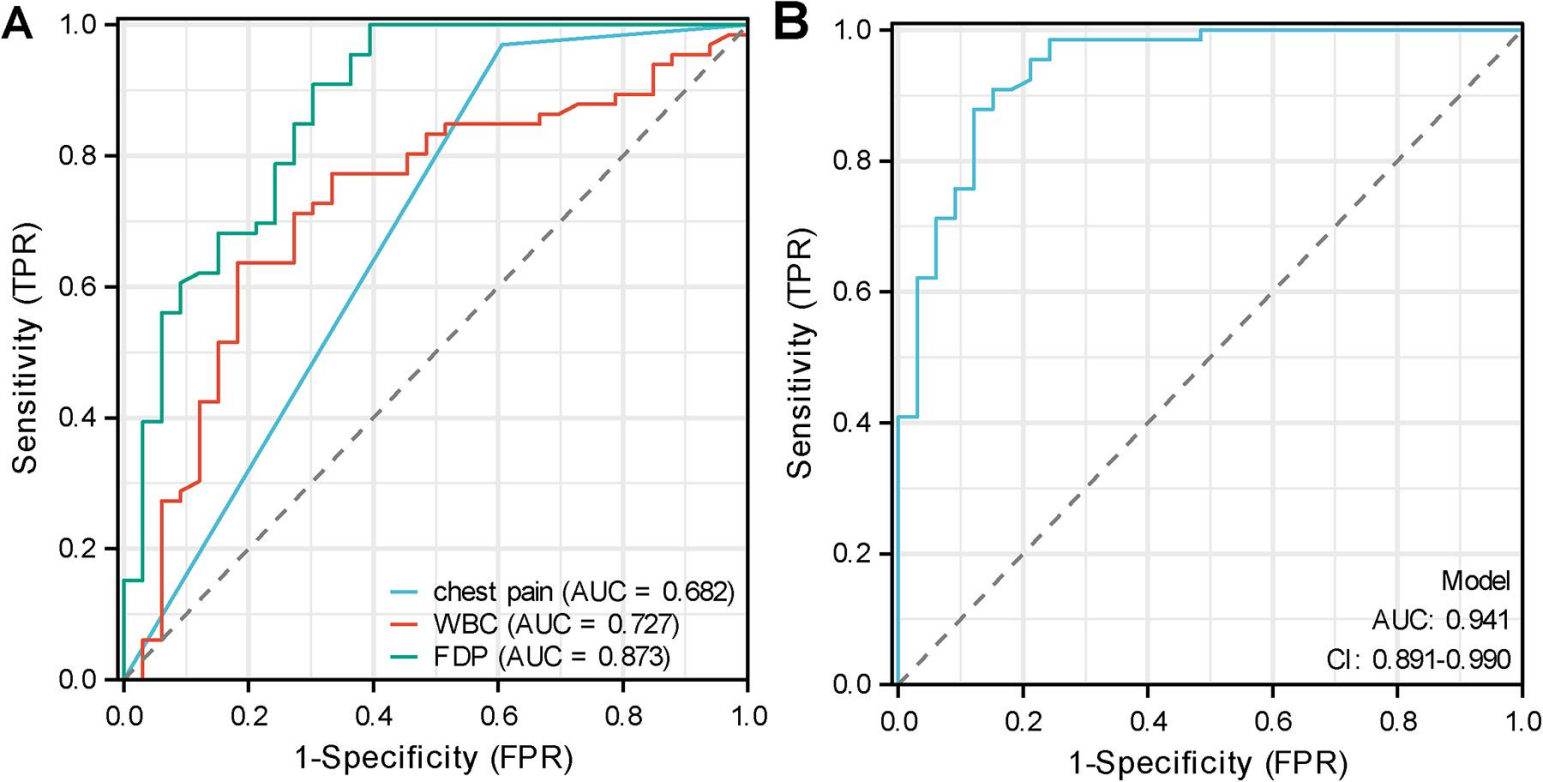
Receiver operating characteristic (ROC) curves for predicting necrotizing pneumonia (NP). **(A)** ROC curves of individual predictors. **(B)** ROC curve of the combined prediction model. All included variables were obtained at the time of prior to the development of NP, ensuring their predictive value.

To evaluate the predictive value of chest pain, WBC, and FDPs for NP, ROC curve analysis was performed (Figure 2 and Table 4). FDPs demonstrated the highest predictive performance (AUC = 0.873, 95% CI: 0.795–0.951), followed by WBC (AUC = 0.727, 95% CI: 0.618–0.835) and chest pain (AUC = 0.682, 95% CI: 0.595–0.769). The optimal cut-off values for FDPs and WBC were 6,585 μg/L and 13.335 × 10^9^/L, respectively, with corresponding sensitivity/specificity of 90.91%/69.70% and 63.64%/81.82%. For chest pain, the sensitivity and specificity were 96.97% and 39.39%, respectively.

**Table 4:** ROC curve analysis of independent predictors for necrotizing pneumonia.

Variables	Cut-off	AUC	95% CI	Sensitivity (%)	Specificity (%)
Chest pain	—	0.682	0.595–0.769	96.97	39.39
WBC (×10^9^/L)	13.335	0.727	0.618–0.835	63.64	81.82
FDPs (μg/L)	6,585	0.873	0.795–0.951	90.91	69.70
Combined model	0.775	0.941	0.891–0.990	87.88	87.88

AUC, area under the curve; CI, confidence interval; WBC, white blood cell count; FDPs, fibrin degradation products. The combined model was constructed using chest pain, WBC, and FDPs. The optimal cut-off values were determined based on the maximum Youden index.

## 4 Discussion

NP is a severe complication of CAP. Although its overall incidence is relatively low, it is characterized by rapid progression, frequent complications, and complex clinical management. In the present study, children with NP exhibited significant differences in clinical manifestations, laboratory parameters, and coagulation profiles compared with those without NP, suggesting that its development is driven by multiple interacting factors. Furthermore, our analysis identified FDPs, WBC, and chest pain as important indicators associated with NP, which may facilitate early identification of high-risk patients and support optimized clinical management.

Previous studies have shown that the most common pathogens associated with NP in children include *Streptococcus pneumoniae*, *Staphylococcus aureus*, *Mycoplasma pneumoniae*, and *Haemophilus influenzae* (10). In addition, other bacterial pathogens such as *Pseudomonas aeruginosa*, *Streptococcus pyogenes*, and *Klebsiella pneumoniae*, as well as viruses including influenza and parainfluenza viruses, have also been implicated in NP (11). Mixed viral–bacterial infections are common and may exacerbate disease severity. Notably, influenza-associated bacterial co-infection has been identified as an important risk factor for NP, possibly due to impaired macrophage function and reduced bacterial clearance (10). In the present study, *Mycoplasma pneumoniae* was the most frequently detected pathogen in children with NP, followed by *Streptococcus pneumoniae*, *Haemophilus influenzae*, and *Staphylococcus aureus*. Compared with previous reports in which Streptococcus pneumoniae and Staphylococcus aureus were the leading pathogens (12), their detection rates were relatively lower in our cohort. This difference may partly reflect the epidemiological predominance of *Mycoplasma pneumoniae* during the study period and the widespread use of pneumococcal vaccination (13). However, the detection of *Mycoplasma pneumoniae* should be interpreted with caution, as it may have represented a co-infecting organism rather than the sole etiological driver of necrotizing pneumonia in some patients. Furthermore, epidemiological data indicate a marked increase in Mycoplasma pneumoniae infections among children in China following the COVID-19 pandemic, particularly since June 2023, with mixed infections being common (14). This trend may be related to increased antimicrobial resistance and altered population immunity in the post-pandemic era. With rising macrolide resistance, *Mycoplasma pneumoniae*-associated pneumonia tends to have a prolonged disease course and greater severity, which may contribute to an increased risk of NP (15–18). These factors may partly explain the recent increase in the incidence of NP.

From a pathophysiological perspective, coagulation dysfunction may play a critical role in the development and progression of NP. FDPs are degradation products of fibrinogen generated through fibrinolysis, including components such as D-dimer, and their elevation generally reflects a hypercoagulable state with secondary hyperfibrinolysis. Inflammation and coagulation are closely interconnected systems in severe infections; inflammatory responses can activate the coagulation cascade, while coagulation abnormalities may, in turn, amplify the inflammatory process (19). Previous studies have demonstrated that coagulation markers, such as FDPs and D-dimer, are significantly elevated in children with severe pneumonia compared with those with non-severe disease, and are closely associated with disease severity and prognosis (20). In addition, in studies of *Mycoplasma pneumoniae* pneumonia and NP, FDPs and D-dimer have also been shown to have predictive value for disease severity and the occurrence of complications (21, 22). In the present study, both FDPs and D-dimer levels were elevated in patients with NP, suggesting the presence of marked coagulation dysfunction and a tendency toward thrombosis. Notably, imaging findings in cases with pulmonary embolism indicated that the locations of thrombus formation corresponded to areas of pulmonary necrosis, further supporting the role of vascular occlusion and ischemic injury in the development of NP.

In addition to coagulation abnormalities, the intensity of the inflammatory response also appears to play a key role in the development of NP. In the present study, WBC was identified as an independent factor associated with NP, indicating that the degree of inflammation is closely related to disease progression. Consistent with our findings, several previous studies have reported that elevated WBC is associated with an increased risk of NP and may serve as an independent predictive marker (23–26). In our cohort, WBC levels were significantly higher in the NP group than in the NNP group, with an optimal cut-off value of 13.335 × 10^9^/L. This threshold was slightly lower than that reported in some previous studies (11), which may be attributable to differences in pathogen distribution and sample size.

From a clinical perspective, chest pain was more frequently observed in the NP group and demonstrated potential predictive value. This symptom may be associated with pleural involvement or an increased extent of pulmonary inflammation and is particularly common in *Mycoplasma pneumoniae* pneumonia. In the present study, the proportion of *Mycoplasma pneumoniae* infection was higher in the NP group, and the incidence of chest pain was significantly greater than in the NNP group, consistent with previous reports (23, 27). Moreover, prior studies have suggested that chest pain may indicate an increased risk of serious complications such as pulmonary embolism (28). In conjunction with the coagulation abnormalities observed in this study, chest pain may partly reflect pulmonary vascular or pleural involvement. Therefore, in children with severe pneumonia presenting with chest pain, clinicians should maintain a high index of suspicion for NP and closely monitor disease progression.

This study has several limitations. First, as a single-center retrospective study with a relatively small sample size, it is subject to potential selection and information biases, which may limit the generalizability of the findings. Second, the relatively long study period may have introduced variability in pathogen distribution and treatment strategies, potentially influencing the results. Third, pathogen testing was not uniformly performed in all patients, which may have led to underdetection or misclassification of infectious agents. Although bronchoalveolar lavage fluid testing improved pathogen detection, the retrospective nature of the study and the complexity of mixed infections limited our ability to determine whether Mycoplasma pneumoniae was the primary etiological pathogen or a concomitant infection in each case. Importantly, all laboratory and clinical variables, except outcome measures, were collected at hospital admission and prior to the development of NP, ensuring that the predictive model variables were obtained before the outcome and avoiding data leakage. However, dynamic changes over time were not evaluated, limiting assessment of their prognostic value throughout the disease course. Although LASSO and multivariable logistic regression were applied, the possibility of model overfitting cannot be entirely excluded. Furthermore, the predictive model developed in this study has not been externally validated, and its stability and clinical applicability require confirmation in multicenter studies with larger sample sizes and prospective designs.

## 5 Conclusion

In summary, this study demonstrated that chest pain, WBC, and FDPs were independently associated with the development of NP in children with severe pneumonia, with FDPs showing the highest predictive value. The combined model significantly improved the identification of NP, highlighting the importance of integrating multiple indicators in risk assessment. From a mechanistic perspective, the interaction between inflammation and coagulation dysfunction, particularly hypercoagulability and microthrombus formation, may contribute to ischemic lung injury and necrosis. Clinically, elevated FDPs and WBC in conjunction with chest pain should raise suspicion for NP and prompt timely imaging evaluation to facilitate early intervention. Nevertheless, these findings require further validation in multicenter, large-scale prospective studies. Future research incorporating dynamic biomarkers may help refine predictive models and explore potential therapeutic strategies.

## Data Availability

The original contributions presented in the study are included in the article/Supplementary Material, further inquiries can be directed to the corresponding author.
